# S100PBP is regulated by mutated *KRAS* and plays a tumour suppressor role in pancreatic cancer

**DOI:** 10.1038/s41388-023-02851-y

**Published:** 2023-10-04

**Authors:** K. Srivastava, K. E. Lines, D. Jach, T. Crnogorac-Jurcevic

**Affiliations:** 1 Centre for Cancer Biomarkers and Biotherapeutics, Barts Cancer Institute, Queen Mary University of London, London, EC1M 6BQ UK; 2 In-Vitro Pharmacology, UCB Pharmaceuticals Ltd, 216 Bath Road, Slough, Berkshire, SL1 3WE UK

**Keywords:** Pancreatic cancer, Oncogenes

## Abstract

We have previously shown that expression of S100PBP, an S100P binding partner, gradually decreases during progression of pancreatic ductal adenocarcinomas (PDAC). Here, we show that loss of S100PBP leads to oncogenic transformation of pancreatic cells; after deregulation of *S100PBP* expression, both in silico and in vitro analyses highlighted alterations of genes known to modulate cytoskeleton, cell motility and survival. Overexpression of S100P reduced S100PBP expression, while co-immunoprecipitation indicated the interaction of S100P with S100PBP-p53-ubiquitin protein complex, likely causing S100PBP degradation. The doxycycline-induced Kras^G12D^ activation resulted in decreased S100PBP levels, while low-dose treatment with HDAC inhibitor MS-275 rescued its expression in both human and mouse PDAC cell lines. This indicates Kras^G12D^ as an upstream epigenetic regulator of S100PBP. Finally, analysis of TCGA PanCancer Atlas PDAC datasets demonstrated poor prognosis in patients with high *S100P* and low *S100PBP* expression, suggesting that S100PBP is a novel tumour suppressor gene with potential clinical utility.

## Introduction

We have previously discovered a novel interacting partner of small Ca^2+^-binding S100P protein, S100PBP (S100P-binding protein) [1], which is almost ubiquitously expressed and shows no homology to any currently known protein. We have also demonstrated that silencing or overexpression of S100PBP leads to increase and decrease of cathepsin Z (*CTSZ*), respectively, and that S100PBP mediates cell adhesion by CTSZ/α_v_β_5_ integrin-specific interaction [2]. Very few additional studies on S100PBP have been reported until now. Integrated bioinformatic analysis of our array data [2] to identify genes and pathways downstream of S100PBP in pancreatic cancer [3] showed its role in miRNA signalling, in cytoskeletal anchoring and protein binding activity, as well as its role in inhibiting migration and invasion. Xie et al. demonstrate S100PBP to be one of the targets of *miR-944*, which is located in the intron of tumour protein p63 gene (*TP63*) and promotes cell proliferation, migration and invasion in cervical cancer [4]. Finally, genome-wide scanning of copy number alterations identified *S100PBP* as one of the genes in three-gene signature which proved to be a reliable biomarker to predict relapse-free survival in post-hepatectomy patients with colorectal cancer liver metastases [5]. However, no other information about this protein is available, and detailed functional roles of S100PBP are currently completely unknown.

In pancreas, S100PBP is expressed in both exocrine and endocrine compart, and is largely confined to the nucleus [1, 2]. However, in pancreatic intraepithelial neoplasia (PanIN), precursor lesions to pancreatic ductal adenocarcinoma (PDAC), it was either expressed in the cytoplasm or was lost. This gradual loss of S100PBP expression during PDAC progression was in stark contrast to increasing levels of pro-oncogenic S100P protein [1, 2]. Given that S100P overexpression is linked with cytoskeletal remodelling and increased motility and invasion [6], we hypothesised that S100PBP could also be implicated in their physiological regulation.

In this study, we therefore aimed to unravel the detailed roles of S100PBP, its interactions with effector molecules which may modulate cell morphology, motility and invasion, and cell survival, all of which could corroborate its potential role of novel tumour suppressor.

## Results

### A role of S100PBP in cell morphology, motility and invasion

**Fig. 1:**
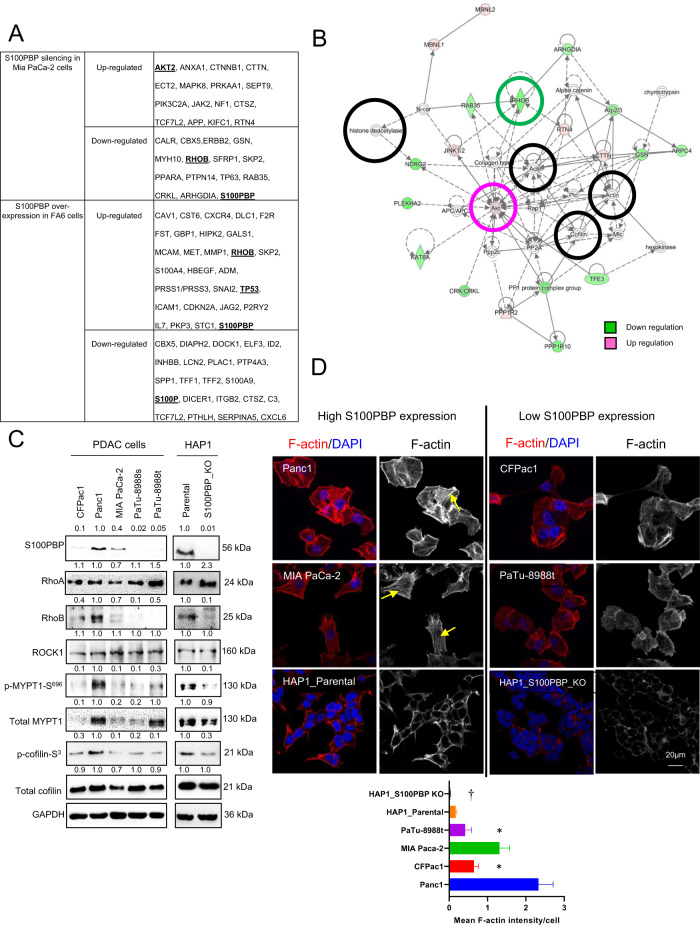
Network analysis highlights the role of S100PBP in modulating cellular movement. Ingenuity Pathway Analysis (IPA) of gene profiling datasets generated in MIA PaCa-2 and FA6 pancreatic cancer cell lines after deregulation of S100PBP expression: **A** Filtered genes involved in cellular movement, and **B** Built relevant networks. **C** Differential expression of S100PBP, RhoA, RhoB, ROCK1, p-MYPT1-S^696^, total MYPT1, p-cofilin-S^3^ and total cofilin in panel of PDAC and HAP1 cells (parental vs. S100PBP_knock out (KO). GAPDH was used as a loading control. Numbers above the Western blots represent the fold changes in protein expression compared with controls (Panc1 and HAP1-Parental) after densitometric analysis. **D** Cellular localisation of F-actin and mean signal intensity/cell in PDAC and HAP1 cell lines. Nuclei are stained by DAPI. Arrows represent stress fibre formation. Scale bar: 20 µm. *N* = 3, mean ± SD, HAP1 S100PBP KO cells vs. HAP1 parental, ^†^*p* < 0.05; low S100PBP expressing PDAC cell lines vs. Panc1 cell line, *p < 0.05.

In silico IPA analysis of our previous transcriptome data of MIA PaCa-2 and FA6 PDAC cell lines after silencing and overexpressing *S100PBP* [2] highlighted the involvement of several signalling networks known to modulate cell morphology, motility and survival, such as RhoB, p53 and AKT signalling (Fig. 1A, B). This was verified in vitro in CML and several PDAC cell lines: the S100PBP protein was highly expressed in Panc1, MIA PaCa-2 and HAP1-parental cells, which also showed elevated expression of RhoB, p-myosin phosphatase1 (MYPT1)-S^696^ and p-cofilin-S^3^, indicating an active RhoB/Rho-kinase (ROCK) signalling (Fig. 1C). In contrast, the cells expressing low levels of S100PBP (CFPac1, PaTu-8988s/t) and HAP1-S100PBP knockout cells showed low levels of these proteins (Fig. 1C), indicating the potential regulatory role of S100PBP in RhoB/ROCK signalling. This was also supported by cellular localisation of F-actin, with an intense F-actin presence in cortical area, alongside presence of stress fibres in cells with high S100PBP, while diminished F-actin cortical staining and loss of stress fibres were observed in cells expressing low or no S100PBP expression (Fig. 1D). Interestingly, this cytoskeletal rearrangement which correlated with S100PBP expression status was also manifested in distinct cellular morphology: while cells expressing high levels of S100PBP appeared to have cobblestone-like polygonal shape with well-defined morphology, the cells expressing low S100PBP appeared to have spindle, elongated shape with poorly defined morphology (Supplementary Fig. 1a).

**Fig. 2:**
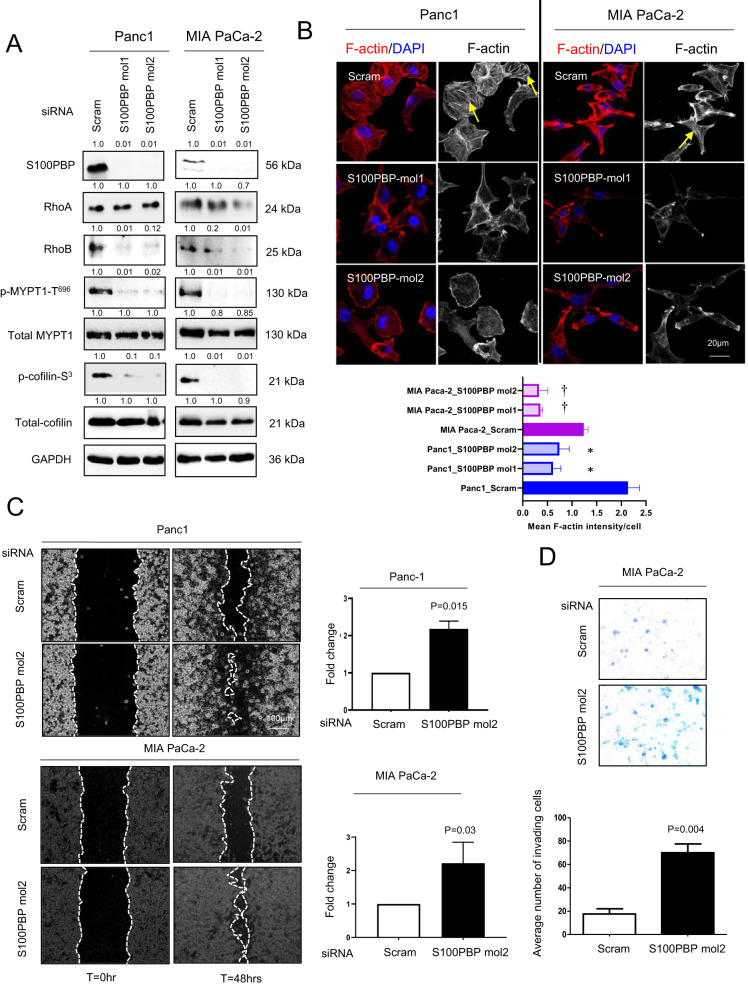
S100PBP silencing disrupt RhoB signalling and increases cell migration and invasion. **A** Expression of S100PBP, RhoA, RhoB, p-MYPT1-S^696^, total MYPT1, p-cofilin-S^3^, total cofilin and control GAPDH after transient silencing of S100PBP by specific siRNA mol1 (S100PBP mol1) and mol2 (S100PBP mol1) in Panc1 and MIA PaCa-2 cell lines. Control cells were transfected by non-target siRNA (scram). Numbers above the Western blots represent the fold changes in protein expression compared with respective controls after densitometric analysis. **B** Cellular localisation of F-actin and mean signal intensity/cell in the abovementioned cell lines. Arrows represent stress fibre formation. *N* = 3, mean ± SD, Panc1 cells after silencing S100PBP compared to Panc1_Scram, *p < 0.05; and MIA PaCa-2_Scram compared to MIA Paca-2 after S100PBP silencing, ^†^*p* < 0.05. **C** Increased rate of cell migration detected by time-lapsed microscopy after S100PBP silencing with siRNA mol2 in Panc1 and MIA PaCa-2 cells. Nuclei are stained by DAPI. **D** Increased invasion of MIA PaCa-2 cells after silencing S100PBP (S100PBP mol2) detected by Matrigel invasion assay: representative Matrigel insert (top panel), number of invading cells (bottom panel).

To further confirm that S100PBP status governs the stability of cytoskeleton, we silenced S100PBP using two independent siRNA molecules (siRNA mol1 and 2) in S100PBP-expressing Panc1 and MIA PaCa-2 cells. This led to lower expression and activity of RhoB marked by low levels of RhoB, p-MYPT1-S^696^ and p-cofilin-S^3^ in S100PBP silenced groups compared to scram (non-target) siRNA-treated control group (Fig. 2A). The low level of RhoB mRNA in HAP1-S100PBP knockout cells and Panc1 cells after transient S100PBP silencing indicates the S100PBP-mediated transcriptional modulation of RhoB (Supplementary Fig. 2a, b, third panel). The localisation of F-actin in Panc1 and MIA PaCa-2 cells after S100PBP silencing was further supportive of these results, with intense F-actin cortical staining alongside stress fibre formations in the control cells, and a weak F-actin cortical staining with loss of stress fibres in S100PBP-silenced cells (Fig. 2B). Similar F-actin distribution in HAP1 cells after S100PBP gene knockout was also shown (Fig. 1D).

We next assessed the functional significance of silencing S100PBP in Panc1 and MIA PaCa-2 cells (only siRNA mol2 was used). The 2D scratch wound assay and time-lapse microscopy demonstrated that S100PBP silencing significantly enhanced the rate of cell migration (Fig. 2C), showing that S100PBP-induced morphological changes are accompanied by changes in cellular motility. Furthermore, S100PBP-silenced MIA PaCa-2 cells also showed significantly higher number of cells invading the Matrigel-coated inserts compared to their matching controls (Fig. 2D); increased invasive capabilities of Panc1 cells after S100PBP silencing we already reported on previously [2]. Thus, silencing of S100PBP potentiated the cell motility and invasion capabilities.

**Fig. 3:**
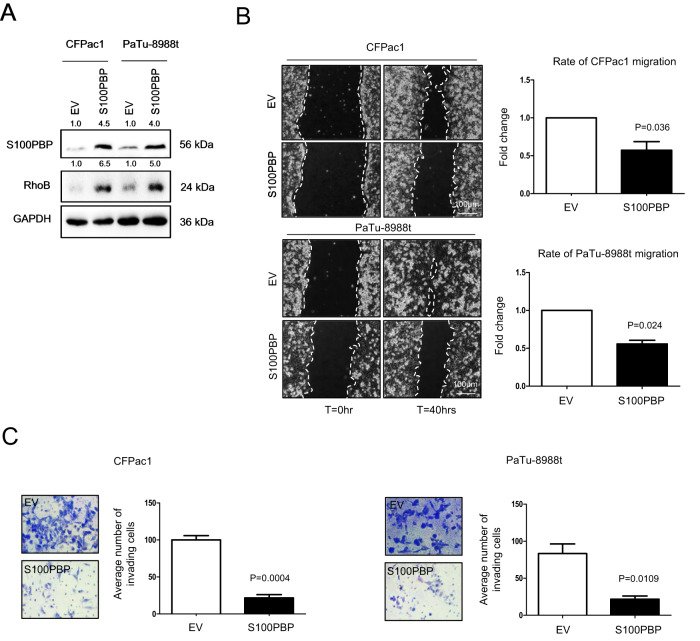
Stable overexpression of S100PBP attenuates cell migration and invasion. **A** Levels of S100PBP and RhoB in CFPac1 and PaTu-8988t cells after stable expression of S100PBP. EV = cells transduced with empty vector, which acted as controls. GAPDH was used as a loading control. Numbers above the Western blots represent the fold changes in protein expression compared with respective controls after densitometric analysis. **B**, **C** Significant decrease in migration and invasion, respectively is shown.

To further ascertain the roles of S100PBP in regulation of cell morphology, motility and invasion, we generated stable S100PBP over-expressing cell lines CFPac1 and PaTu-8988t, which endogenously express low levels of S100PBP (Fig. 3A and Supplementary Fig. 3a). Such engineered cells expressed high levels of RhoB protein (Fig. 3A) which coincided with their distinct round, compact, cobblestone-like polygonal morphology compared to the empty vector (EV) control cells, which retained elongated shape and spindle morphology (Supplementary Fig. 1b). In addition, the high S100PBP expressing population showed a significantly lower rate of both cell migration and invasion (Fig. 3B, C). Taken together, these results confirm the role of S100PBP in (negative) regulation of motility and invasion.

### S100PBP interacts with p53 and is involved in apoptosis regulation via AKT signalling

**Fig. 4:**
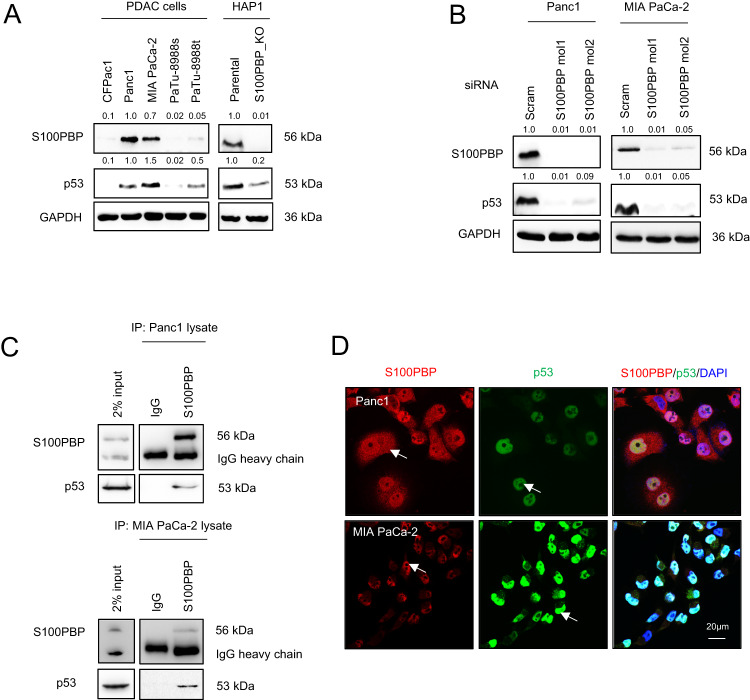
Interaction of p53 and S100PBP proteins. **A** Expression of S100PBP and p53 in panel of PDAC cells and parental vs. S100PBP_knock out (KO) HAP1 cells (parental vs. S100PBP_knock out (KO)) before and (**B**) after transient silencing by siRNA molecules (Scram, S100PBPmol1 and mol2) in Panc1 and MIA PaCa-2 cells. GAPDH was used as a loading control. Numbers above the Western blots represent the fold changes in protein expression compared with respective controls (Panc1, HAP1-Parental and Scram siRNA transfected cells) after densitometric analysis. **C** Co-immunoprecipitation of S100PBP and p53 proteins in IP samples from Panc1 (top panel) and MIA PaCa-2 (bottom panel) lysates. Rabbit-IgG acted as internal control; input sample was 2% of whole cell lysates. **D** Cellular localisation of S100PBP and p53 in Panc1 and MIA PaCa-2 cells. Nuclei are stained by DAPI. Arrows represent sub-cellular localisation of respective proteins; the co-localisation of both proteins suggests that they interact in the nucleus.

IPA highlighted *TP53* and *AKT* as additional effector genes involved in cell movement after deregulation of *S100PBP* (Fig. 1A). Interestingly, the endogenous levels of p53 in PDAC and HAP1 cells mirrored the pattern of their S100PBP expression (Fig. 4A). Furthermore, the transient S100PBP gene silencing in Panc1 and MIA PaCa-2 cells reduced both the mRNA and protein levels of p53 (Fig. 4B and Supplementary Fig. 2b, middle panel), thus indicating the involvement of S100PBP in modulation of p53 expression. As several S100 proteins bind to and interact with p53 [7, 8], we wanted to establish if p53 also interacts with S100PBP. Indeed, the co-immunoprecipitation studies using whole cell lysates of Panc1 and MIA PaCa-2 cells indicated that these two proteins can also interact (Fig. 4C). In these two cell lines, p53 was shown to be confined to the nucleus (the intense nuclear localisation of p53 was also observed in stable S100PBP over-expressing CFPac1 cells, Supplementary Fig. 4a), suggesting that this is where S100PBP and p53 interact (Fig. 4D).

**Fig. 5:**
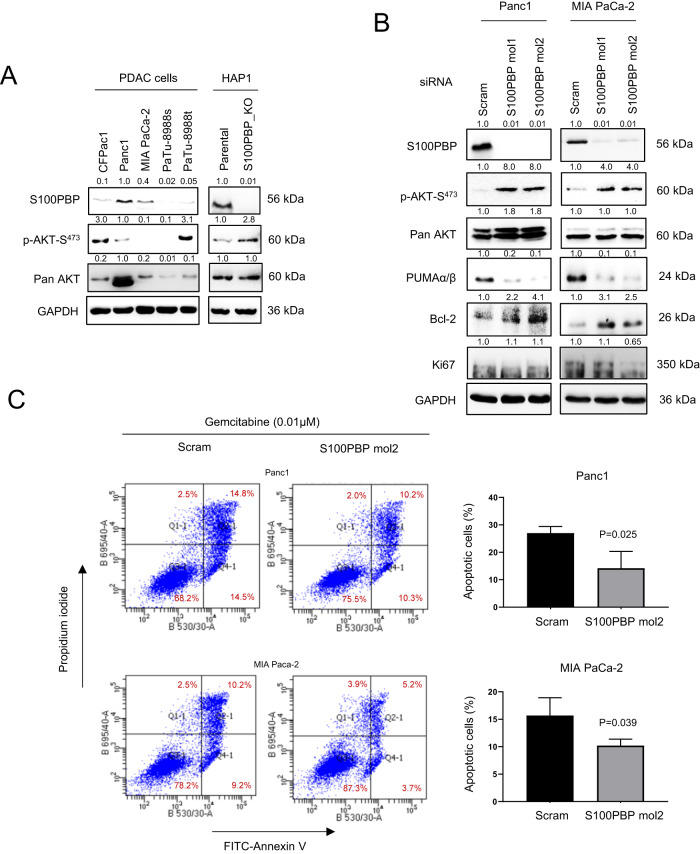
Inverse correlation between pro-survival AKT and S100PBP proteins. **A** Expression of S100PBP, p-AKT-S^473^ and pan AKT in panel of PDAC and HAP1 cells (parental vs. S100PBP_knock out (KO)) before and (**B**) after transient silencing of S100PBP by specific siRNA mol1 and mol2 in Panc1 and MIA PaCa-2 cell lines. The differential protein expression of PUMAα/β and Bcl-2 are also shown. No changes in Ki67 were seen. GAPDH was used as a loading control. The scram (non-target) siRNA transfected cells were treated as respective controls. Numbers above the Western blots represent the fold changes in protein expression compared with respective controls (Panc1, HAP1-Parental and Scram siRNA transfected cells) after densitometric analysis. **C** Increased percentage of apoptotic cells detected by FITC-Annexin V/Propidium iodide flow cytometry after S100PBP silencing (mol2) in Panc1 and MIA PaCa-2 cells followed by gemcitabine (0.01 µM) treatment for 96 h was observed.

**Fig. 6:**
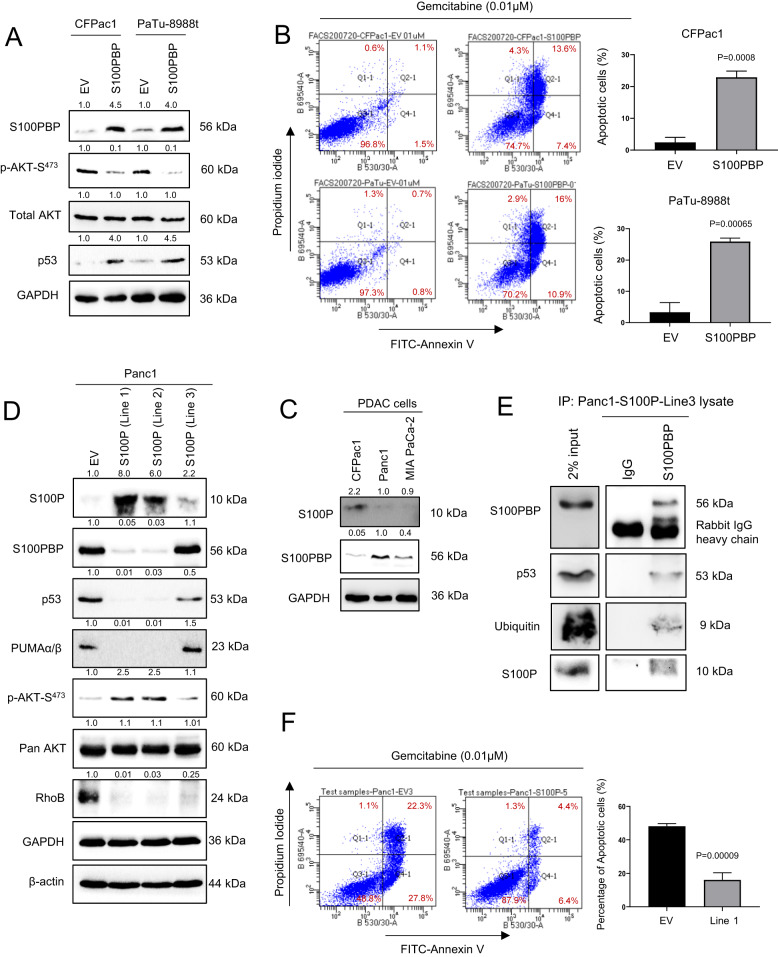
S100PBP attenuates cell survival, while S100P downregulates S100PBP and increases survival of cancer cells. **A** Expression of S100PBP, p-AKT-S^473^, pan AKT, and p53 in CFPac1 and PaTu-8988 cells stably expressing S100PBP protein. Cells transduced with empty vector (EV) were treated as respective controls. GAPDH was used as a loading control. **B** Significant increase in percentage of apoptotic cells detected by FITC-Annexin V/Propidium iodide flow cytometry in stably expressing S100PBP cells after gemcitabine (0.01 µM) treatment for 96 h is shown. **C** Inverse expression of S100P and S100PBP in PDAC cell lines (CFPac1 and Panc1 (control) and MIA Paca-2). GAPDH was used as a loading control. **D** Expression of S100P, S100PBP, p53, PUMAα/β, p-AKT-S^473^, pan AKT and RhoB in three Panc1 cell lines (Line 1, 2 and 3) after stable overexpression of S100P. Panc1 cells transduced with empty vector (EV) were used as controls. GAPDH and β-actin were loading controls. The numbers above the Western blots represent the fold changes in protein expression compared with respective controls after densitometric analysis. **E** Immunoprecipitation (IP) of S100PBP protein and subsequent detection of p53, ubiquitin and S100P proteins in IP samples from Panc1-S100P Line3 lysates. Rabbit-IgG acted as internal control and input sample was 2% of whole cell lysates. **F** Significant decrease in percentage of apoptotic cells detected by FITC-Annexin V/Propidium iodide flow cytometry in Panc1-S100P (Line 1) cells after gemcitabine (0.01 µM) treatment for 96 h.

We next studied the potential role of S100PBP in modulation of AKT signalling as suggested in Fig. 1B. We observed the activation of AKT pathway, marked by upregulation of p-AKT-S^473^ in cells with low S100PBP expression (Fig. 5A). Consistent with these results, the transient silencing of S100PBP expression in Panc1 and MIA PaCa-2 cells decreased the levels of p53 (Fig. 4B) and its transcriptional target, pro-apoptotic PUMAα/β, while activating AKT and upregulating anti-apoptotic Bcl-2 protein (Fig. 5B). Since Ki67 levels remained unaffected in these cells, the activation of pro-survival AKT signalling appeared to be independent of its cell proliferation role (Fig. 5B and Supplementary Fig. 4a). The functional significance of AKT activation was studied by FITC-Annexin V flow cytometry after silencing S100PBP in Panc1 and MIA PaCa-2 cells, before treating these cells and their respective controls (Scram) with gemcitabine (0.01 µM) for 96 h. The significant reduction in the percentage of apoptotic cells in the S100PBP-silenced population is shown on Fig. 5C, implicating S100PBP in apoptotic modulation via AKT signalling. Using PDAC cells with ectopic expression of S100PBP, we further observed that cells with high S100PBP expression also expressed elevated levels of p53 protein, while alleviating pAKT-S^473^ levels (Fig. 6A). The pro-survival role of S100PBP was further confirmed by treating the control CFPac1 and PaTu-8988t cells and their S100PBP-expressing counterparts with 0.01 µM gemcitabine for 96 h. The flow cytometry data indicated significant increase in the percentage of apoptotic cells with high S100PBP levels (Fig. 6B). The overactivity of pro-apoptotic caspase-3, marked by high levels of cleaved caspase-3 and presence of cleaved PARP seen in these cells further suggested the role of S100PBP in increased chemosensitivity (Supplementary Fig. 4b).

Given that several S100 proteins, including S100P, play a pivotal role in posttranslational modification, stabilisation and cellular trafficking of p53 [8, 9] and that the expression of S100P increases with concomitant depletion of S100PBP during PDAC development [2], we investigated the role of S100P in modulating S100PBP expression. Similarly to what we have shown previously in histological analysis, Western blot data showed that the high S100PBP-expressing cells Panc1 and MIA PaCa-2 express low levels of S100P, in contrast to CFPac1 cells with high S100P expression (Fig. 6C). To further investigate this, we established three Panc1 cell lines with stable expression of S100P: Line 1 and 2 had high levels, and Line 3 low levels of S100P (Fig. 6D). Analysis of these three cell lines and EV control Panc1 cells showed low levels of S100PBP, p53, PUMAα/β and RhoB, and activated AKT in the Lines 1 and 2 (Fig. 6D). Interestingly, the cell lines stably expressing S100P showed distinct morphological changes: while the control cells exhibited cuboidal shape with cobblestone-like morphology, Panc1 lines 1 and 2 with high S100P showed elongated shape and spindle morphology (Supplementary Fig. 1c). Furthermore, the S100PBP transcript expression in these cells did not change significantly, suggesting that changes in protein levels were not due to transcriptional regulation (Supplementary Fig. 5, two upper panels). Panc1 line 3, which expresses low levels of S100P and high level of S100PBP protein (Fig. 6D) was utilised for the immunoprecipitation experiments, which confirmed the S100PBP-p53 protein interaction observed earlier. Furthermore, additional co-immunoprecipitation of S100P and ubiquitin in the same sample and their presence in S100PBP-p53 complex (Fig. 6E) potentially indicated the targeting of S100PBP and/or p53 for degradation. This suggests that the interaction of S100P and S100PBP (and p53) is highly dynamic and transient, and could explain somewhat counteractive finding of inverse expression of S100P and S100PBP in cells in vitro and in tissue sections, as reported previously [1, 2].

The functional significance of the AKT activation in Panc1 cells expressing high S100P levels was studied by treating control and line 1 cells with 0.01 µM gemcitabine for 96 h followed by FITC-Annexin V apoptotic assay. The results indicated a significant reduction in percentage of apoptotic line 1 cells compared to the control population (Fig. 6F), confirming the role of S100P in enhancing chemoresistance.

### Kras^G12D^ regulates S100PBP via epigenetic mechanism

**Fig. 7:**
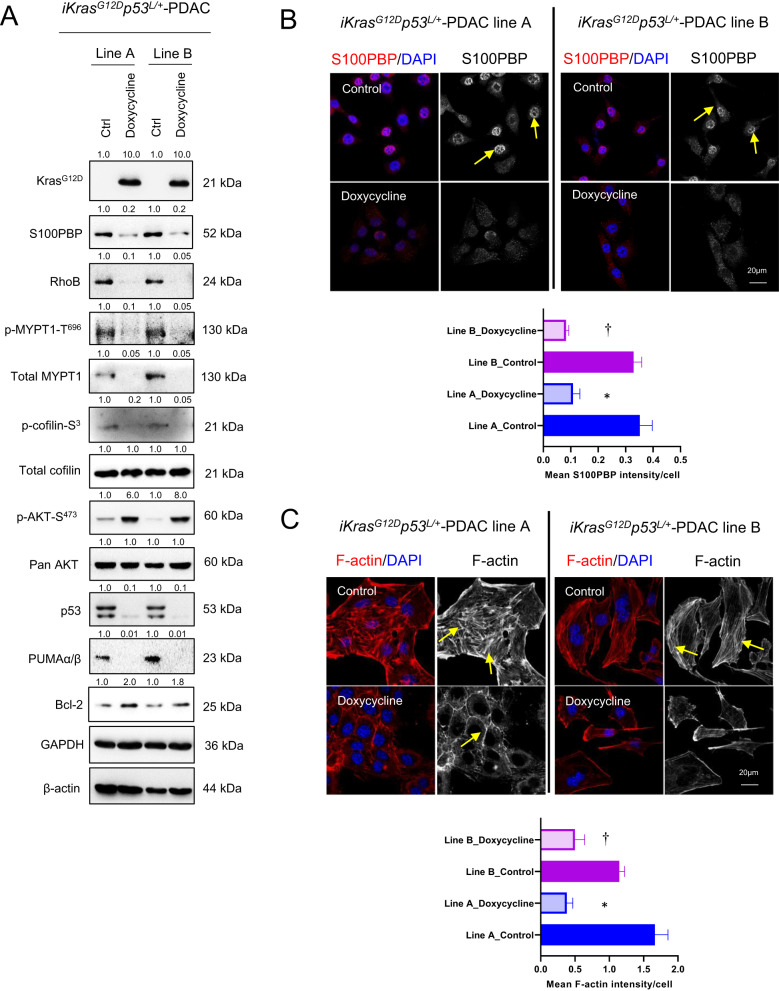
Kras^G12D^ activation downregulates S100PBP while activating AKT signalling. **A** Differential expression of Kras^G12D^, S100PBP, RhoB, p-MYPT1-S^696^, total MYPT1, p-cofilin-S^3^, total cofilin, p-AKT-S^473^, pan AKT, p53, PUMAα/β and Bcl-2 proteins in two iKras mouse PDAC cell lines (A and B) after doxycycline (1 µg/mL) treatment for 24 h. GAPDH and β-actin were used as loading controls. **B** Translocation of S100PBP (arrows) from nuclei (DAPI) in untreated (Control) iKras cells to the cytoplasm in Doxycycline treated cells. **C** Changes in F-actin and mean signal intensity/cell in the abovementioned cell lines. Arrows represent thick cortical staining and stress fibre formation. *N* = 3, mean ± SD, Line A_Doxycycline compared to Line A_Control, *p < 0.05; Line B_Doxycycline compared to Line B_Control, ^†^*p* < 0.05. The numbers above the Western blots represent the fold changes in protein expression compared with respective controls after densitometric analysis.

Mutant *KRAS* is known regulator of various S100 proteins in many cancers [10, 11], and is an earliest driver of PDAC development; we thus wanted to establish if mutant *KRAS* plays a role in modulating the expression of S100PBP, especially since total KRAS levels remained largely unchanged after stable expression of S100PBP in CFPac1 and PaTu-8988t cells (Supplementary Fig. 3b). We utilised doxycycline-inducible Kras^G12D^ PDAC mouse cell lines (*iKras*^*G12D*^*p53*^*L/+*^) [12] to study the effects of persistent activation of mutant *KRAS* on S100PBP expression. Kras^G12D^ activation resulted in downregulation of S100PBP (Fig. 7A). In addition, upon Kras^G12D^ activation, the translocation of S100PBP to the cytoplasm was seen (Fig. 7B), mimicking the data we observed in human PanIN lesions [1, 2]. Taken together, these results suggest the upstream regulatory role of mutated Kras^G12D^ on S100PBP.

We further show that Kras^G12D^ activation also decreased the levels of RhoB, p-MYPT1-S^696^ and p-cofilin-S^3^, thereby indicating attenuated RhoB signalling axis (Fig. 7A). Furthermore, weak and disrupted F-actin cortical staining was also seen after Kras^G12D^ activation, which showed intense F-actin cortical presence along with stress fibre formations traversing across the cell surface (Fig. 7C). With reorganisation of the cytoskeleton, Kras^G12D^ activation also resulted in altered cellular morphology from round and cobblestone-like shape in control population to elongated and spindle shape in Kras^G12D^-activated population (Supplementary Fig. 1d). Finally, Kras^G12D^ activation also led to the depletion of p53 and PUMAα/β proteins, while increasing Bcl-2 levels and activated pro-survival AKT signalling (Fig. 7A). Taken together, these results substantiated S100PBP as a downstream target of mutated Kras^G12D^, indicating that its loss could be pivotal for PDAC development and progression.

**Fig. 8:**
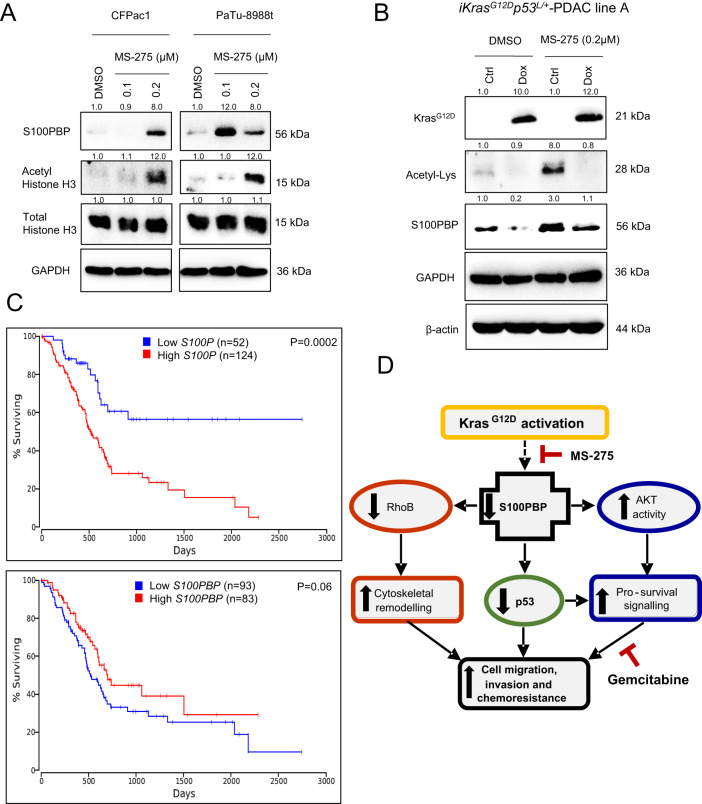
Inhibition of HDAC activity rescues S100PBP expression. **A** Expression of S100PBP, Acetyl histone H3 and total histone H3 in CFPac1 and PaTu-8988t cell lysates after treatment with 0.1 or 0.2 µM MS-275 or DMSO vehicle (control) for 72 h shows rescued S100PBP expression in these cell lines. **B** Expression of Kras^G12D^, S100PBP and Acetyl-Lysine in iKras mouse PDAC cell lysates after 24 h pre-treatment with 0.2 µM MS-275 followed by treatment with doxycycline (1 µg/ml) for further 24 h to confirm the Kras^G12D^-mediated epigenetic modification of S100PBP. GAPDH and β-actin were used as loading controls. The numbers above the Western blots represent the fold changes in protein expression compared with respective controls after densitometric analysis. **C** Kaplan–Meier survival plots using TCGA PanCancer Atlas dataset from 176 pancreatic adenocarcinoma patients. Patients with high S100P and low S100PBP expression had worse survival. **D** Schematic representation of Kras^G12D^/S100PBP signalling axes in PDAC cells.

Since the over-activity of class-I histone deacetylases (HDACs) modulated by mutant KRAS has been reported in PDAC previously [13, 14], and HDAC was also highlighted in our IPA signalling network shown on Fig. 1B, we tested the potential role of HDAC in regulation of S100PBP expression. The treatment of CFPac1 and PaTu-8988t cells with low doses of class-I HDAC inhibitor, MS-275/Entinostat [15], rescued the expression of S100PBP and acetyl histone H3 in a dose dependent manner (Fig. 8A). Additionally, the pre-treatment of Kras^G12D^ PDAC mouse cell line-A with 0.2 µM MS-275 also increased the levels of S100PBP and acetyl lysine (Fig. 8B). While doxycycline-mediated activation of Kras^G12D^ suppressed S100PBP, pre-treatment with 0.2 µM MS-275 largely abated the effects of Kras^G12D^ overactivation by rescuing and maintaining S100PBP levels similar to untreated controls (Fig. 8B). These results indicate that Kras^G12D^-mediated regulation of S100PBP is, at least partially, modulated via epigenetic mechanism.

### High expression of S100P and low expression of S100PBP correlate with poor prognosis

Finally, to establish the potential clinical relevance of *S100P* and *S100PBP*, we studied TCGA PanCancer Atlas dataset on 176 pancreatic adenocarcinoma patients and generated Kaplan–Meier survival plots (Fig. 8C). It is evident that patients with high *S100P* and low *S100PBP* expression have decreased survival.

## Discussion

In this study we report novel tumour suppressive functions of S100PBP, including modulation of cytoskeletal reorganisation, cell morphology, motility and invasion. We show that the underlying mechanisms involve a small GTP-binding protein RhoB and its effector Rho-kinase (ROCK)-1/2 (proxies for this being phosphorylated levels of MYPT-1 and cofilin proteins seen in our data), which are well known to balance the stabilisation/destabilisation of filamentous (F)-actin and modulate the cytoskeleton-dependent processes such as cell shape and motility [16, 17]. We have previously observed deregulation of p-cofolin-S^3^, which inhibits actin polymerisation and cortical F-actin localisation after overexpression of S100P in PDAC cells; S100P is known to be critically involved in regulation of motility and invasion [6]. We have now expanded the study to its binding partner, S100PBP, and demonstrate distinct changes in the F-actin profile marked by the disappearance of stress fibres and/or weak F-actin cortical presence in cells endogenously expressing low S100PBP or after S100PBP gene silencing. Similar morphological changes, alongside enhanced motility and invasion in cells with low F-actin polymerisation and cortical presence have been described previously [18–20]. As these changes match the pattern of RhoB expression and activity—downregulation of RhoB promotes cell migration and invasive behaviour [21, 22], and we demonstrate a direct correlation between RhoB and S100PBP expression in both PDAC and CML cells, this indicates that S100PBP plays an important role in modulating cytoskeleton, cell morphology, motility and invasion via RhoB signalling.

Unlike other members of Rho family, like RhoA and RhoC, which play oncogenic roles [17, 23], RhoB has been shown to suppress tumorigenesis in pancreatic and other cancers [21, 24–26]. Furthermore, in RhoB-deficient mice the number of Ras-induced tumours increased compared to wt-mice [27], and loss of RhoB led to increased metastatic dissemination of lung cancer cells, which was mediated by AKT [21]. This was further substantiated in study by Jiang et al., who showed that oncogenic Ras downregulates RhoB expression by a PI3K/AKT signalling [28]. Finally, low RhoB expression in human PDAC samples [24], which mimics the behaviour of S100PBP described in our previous study [2], firmly implicates S100PBP in the same RhoB signalling circuitry and suggests its tumour suppressive functions.

The role of p53 in modulating cell morphology and motility by supressing cell polarisation, formation of protrusions and spreading was shown previously [29–32]. In addition, several studies have demonstrated the role of p53 in suppressing cell migration and metastasis [33, 34]. Interestingly, these ‘atypical’ roles of p53 are largely mediated via controlling actin cytoskeletal organisation through Rho signalling [35, 36], which is corroborated by data in the present study. In PDAC, p53 is a well-known tumour suppressor: a loss of its functions is critical in dysregulation of cell cycle checkpoints and apoptosis, resulting in acceleration of PDAC progression and metastatic formation in both mouse models of PDAC as well as in pancreatic cancer patients [12, 37–40].

Interestingly, our data suggest that S100PBP both regulated *TP53* at the transcription level, and also interacted with p53 in the nucleus. S100P and several other members of S100 family were previously shown to bind to p53 and interfere with its oligomerisation, stabilisation and cellular trafficking, thus regulating its activity [7–9]. We now demonstrate for the first time that S100PBP also binds to p53, and show the existence of S100PBP-p53-S100P-ub protein complex, which could be pivotal in destabilisation and/or degradation of both S100PBP and p53. While further experiments are needed to delineate the ubiquitination pathway and proteasomal degradation machinery regulating the stability of these two proteins, it is tempting to speculate that (as suggested by loss of S100PBP in PanIN progression seen in histological analysis of PDAC tissues) this is also happening in vivo during PDAC development. Loss of S100PBP would ‘free’ S100P, a highly invasive protein, which will abrogate p53 function, leading to early metastatic spread of PDAC cells. Oncogenic role of S100P after binding and inactivation of p53 contributing to outgrowth of aggressive tumour cells has previously been highlighted [9].

Loss of p53 expression is known to accompany elevated levels of endogenous PI3K (phosphoinositide 3-kinase) and AKT activity [35, 41]. This AKT overactivation has a major role in growth signal autonomy, inhibition of apoptosis and chemoresistance, and is frequently disturbed in many cancer types [42, 43]. In PDAC, AKT is overexpressed by 50%, and its overexpression is well known to be a negative prognostic factor [44, 45]. We observed a positive and an inverse correlation between AKT activation (proxy being pAKT-S473 levels) and S100P/S100PBP expression, respectively, in all studied PDAC cell lines. The ectopic expression of S100PBP in PDAC cells decreased AKT activation and chemoresistance to Gemcitabine, while opposingly, S100PBP silencing in MIA PaCa-2 and Panc1 cells (low S100P) led to increased AKT activity, suggesting the presence of AKT-mediated intrinsic chemoresistance in PDAC cells with low S100PBP expression. This is in line with previously reported finding in Panc1 cells, where inhibition of phosphorylated FAK lead to reduction in phosphorylated AKT, resulting in Gemcitabine-induced cytotoxicity [46].

Given that activating mutations in *KRAS* are initial drivers of PDAC, we hypothesised that they may also directly or indirectly affect the S100PBP signalling. Indeed, Kras^G12D^ activation in mouse cell lines from inducible oncogenic *KRAS* model of PDAC [12] led to downregulation of S100PBP protein and triggered its nuclear-to-cytoplasmic translocation. In addition, the downstream effects of Kras^G12D^ activation on RhoB, AKT and p53 signalling axes mirrored the in vitro data obtained after S100PBP silencing and thus provided mechanistic evidence of KRAS as an upstream regulator of S100PBP signalling axes, as illustrated in Fig. 8D.

Several studies in various cancers, including pancreatic, have also indicated the role of overactivated *KRAS* in supressing the pro-apoptotic and tumour suppressor genes by enhancing the epigenetic modifications of histones [47–50]. The elevated expression and activity of class-I HDACs have been previously reported in PDAC [13, 51]. We here show that the treatment with a class-I specific HDAC inhibitor MS-275 rescued the expression of S100PBP in vitro by, at least partially, attenuating the Kras^G12D^-evoked suppression of S100PBP. This demonstrates a role of epigenetic mechanism in S100PBP regulation, as shown previously for S100P [52]. HDAC inhibitors like vorinostat have shown promising outcomes by augmenting cell death and supressing advanced pancreatic (and other) cancers [47, 49, 50, 53], and our data now implicate S100PBP in the mechanisms of their actions. Further studies with individual members of class-I HDACs, which may have a direct or indirect role in the epigenetic regulation of S100PBP and exert potential therapeutic benefit, are now warranted.

Taken together, in the present study we demonstrate a novel tumour suppressive role of S100PBP via its regulation of cellular morphology, motility, invasion and pro-survival capabilities. We implicate S100PBP as a novel intermediate and a critical signalling molecule in Kras/AKT/RhoB/p53 signalling axes and suggest its epigenetic regulation. Given that the high expression of *S100P* and low expression of *S100PBP* correlate with poor survival in PDAC patients, this study highlights the potential clinical relevance of S100PBP in pancreatic cancer.

## Material and methods

### Tissue culture

Cell lines from human PDAC (CFPac1, Panc1, MIA PaCa2, PaTu-8988s/t), human chronic myeloid leukaemia (CML; HAP1-parental and HAP1-CRISPR-Cas9 S100PBP knockout cells purchased from Horizon Discovery, UK) and doxycycline-inducible Kras^G12D^ mouse PDAC cell lines [12] were cultured in their respective media with 10% FBS and Pen/Strep in humidified tissue culture incubators. The mouse PDAC cell lines were treated with doxycycline (100 μg/ml) for 48 h before protein and RNA analysis. The identity of all the cell lines used were verified by short tandem repeat profiling. The phase contrast images of these cells were captured using ×10 magnification on light microscope (Olympus, Germany).

### Western blotting

The differential protein expressions between cell lines with differing levels of S100PBP expression, and after S100PBP gene manipulation were studied as described previously [54]. Briefly, 50 μg whole cell lysate was resolved by SDS-PAGE using 7.5%–15% polyacrylamide gels before blotting onto nitro cellulose membrane (0.2 μm pore size, GE Health Sciences, UK). The target proteins were detected by antibodies listed in Supplementary Table 1, and visualised by chemiluminescence substrate (Millipore, UK). The protein expression was quantified by densitometric analysis using ImageJ software and the values representing fold changes compared to the control groups are presented above the bands across all Western blots.

### Real-time PCR

qPCR was conducted as previously described [54]. Briefly, the total RNA was harvested from cell pellets by Tripure reagent (Roche, USA) before quantification. Two μg of RNA was used for single strand synthesis by single strand synthesis kit (Roche, USA) before setting qPCR reaction using primers detailed in Supplementary Table 2 and SYBr green reagent (Applied Biosciences, UK) on ABI 7500 real-time machine (Applied Biosciences, UK).

### Immunofluorescence

The cellular localisation of filamentous Actin (F-actin) was conducted by detection and visualisation of Rhodamine-labelled phalloidin (Life Technologies, UK) as previously described [55]. The quantification was performed using Adobe Photoshop 2021 software and values graphically presented as mean F-actin intensity/cell below each image. The other cellular proteins were visualised using LSM710 confocal microscope (Zeiss, Germany) as described previously [54, 56].

### Cell migration and invasion assays

The relative rate of cell migration was studied by culturing the cells (0.5 × 10^6^) in 2D on collagen-I coated 6-well tissue culture plates before treatment with mitomycin C (2 μg/ml) for 2 h and creation of similar size scratch wounds as previously described [56]. The cell motility and wound closure were recorded by capturing scratch wound images using 10X magnification on time-lapsed microscope (Axio-Vision, UK) kept in humidified tissue culture cabinet. For 2D cell invasion assays, the cells (5 × 10^4^) suspended in serum-free DMEM were seeded on Matrigel-coated transwell inserts (0.8 μm pore size) kept in 24-well tissue culture plates before their incubation for 48 h. The cells which passed through the pores were fixed in 100% methanol before their visualisation by Giemsa blue staining. The cells were counted from five representative images/insert captured by ×20 magnification on light microscope (Olympus, Germany).

### Transient gene silencing

Fifty nM siRNA was used to silence S100PBP in cells (Panc1 and MIA PaCa2). Briefly, 0.5 × 10^6^ cells were seeded in tissue culture dishes before treatment with either scram (non-target) or two independent siRNA molecules against human S100PBP (Mol1: AUGGUGGUUCACACAAG UCAA, Mol2: CUGUGUGAGUAAUGCAUUCUA; Qiagen, UK) mixed with RNAiMax transfection reagent (Life Technologies, UK). Fresh medium was replaced after 16 h, and cells were harvested for protein and RNA analysis after 72 h of transfection. A fraction of cells from matching population were cultured on glass coverslips in their respective medium for cellular localisation studies.

### Stable gene over-expression

The stable expression of S100PBP was conducted in low S100PBP-expressing cells (CFPac1 and PaTu-8988t) by using expression vector pCMV-Tag2B carrying full length S100PBP gene and matching empty vector (EV) as previously described [1, 2]. Similarly, the Panc1 cell lines with stable expression of S100P were established using pcDNA3.1 carrying full length S100P. Briefly, cells (0.2 × 10^6^/well) seeded in 6-well tissue culture plates were treated with a mixture of plasmid and FuGene6 transfection (Promega, UK) reagent for 48 h. The transfected cells were selected by treatment with 1.1 mg/ml geneticin (G418 sulfate) antibiotic (InvivoGen, UK) for 3 weeks. The mRNA and protein analyses were conducted to confirm the stable expression of genes in the respective cell lines.

### Co-immunoprecipitation

The protein-protein interactions were studied by co-immunoprecipitation. Briefly, 4 μg rabbit polyclonal antibodies (anti-S100PBP and IgG) were crosslinked with protein G-tagged Dynabeads (Life Technologies, UK) according to the manufacturer’s instructions. The antibody-Dynabeads complexes were incubated with the whole cell lysates (15 × 10^6^ cells lysed in NP-40 buffer) under constant rotation for 24 h at 4 °C. A magnet was used to separate antigen-antibody-Dynabeads complex from the lysates, before non-denaturing elution of antibody-antigen complex. Laemmli buffer (Sigma-Aldrich, UK) was added to the eluted sample and input (2% whole cell lysate) sample before visualising proteins by Western blotting.

### In silico analysis

The Affymetrix gene expression profiling was conducted using RNA isolated from MIA PaCa-2 and FA6 cells after deregulation of S100PBP expression as described previously [2]. These data have been deposited to the National Center for Biotechnology Information’s Gene Expression Omnibus (https://www.ncbi.nlm.nih.gov/geo, accession number GSE35199).

The generated datasets were analysed using Ingenuity Pathway Analysis (IPA) (Qiagen, UK) to build networks of inter-linked signalling molecules. The Cancer Genome Atlas (TCGA) PanCancer Atlas datasets for PDAC (*n* = 177) were used to conduct analysis using the cBioPortal for cancer genomics website (https://www.cbioportal.org/) and the Protein Atlas website (https://www.proteinatlas.org/).

### Flow cytometry

The quantification of cells undergoing apoptosis was conducted by FITC Annexin V apoptotic detection assay kit (BD Biosciences, USA). Briefly, 10^5^ cells/well were seeded on collagen-I coated 6-well plates before 72 h treatment with gemcitabine (0.01 µM). The medium and trypsinised cells were centrifuged at 500 × *g* for 5 min (Thermo Scientific, UK) to form cell pellets, which were resuspended in 1x binding buffer containing FITC-labelled annexin V and propidium iodide (PI). The early and late apoptosis detection was conducted by flow cytometer LSR Fortesa-3 and analysed by FACS DIVA software (BD Biosciences, USA).

### Statistical analysis

All experiments were performed three times unless indicated otherwise. Data are presented as mean ± SD. Statistical analysis was performed using Student’s *t* test and one-way analysis of variance (ANOVA) followed by Dunnet’s post hoc analysis where more than two groups were compared; GraphPad Prism v8 software was utilised. The *p* < 0.05 was considered to be significant.

### Supplementary information

Supplementary figures

Supplementary Table 2

## Data Availability

The data generated during and/or analysed during the current study are available from the corresponding author on reasonable request.
