# Supplementary material for: S100PBP is regulated by mutated KRAS and plays a tumour suppressor role in pancreatic cancer
Source: Oncogene. 2023 Oct 4;42(46):3422–34. doi: 10.1038/s41388-023-02851-y (PMC10638088; doi:10.1038/s41388-023-02851-y)
Supplement: Supplementary file 1 — Supplementary figures [file 41388_2023_2851_MOESM1_ESM.pptx]

## Slide 1
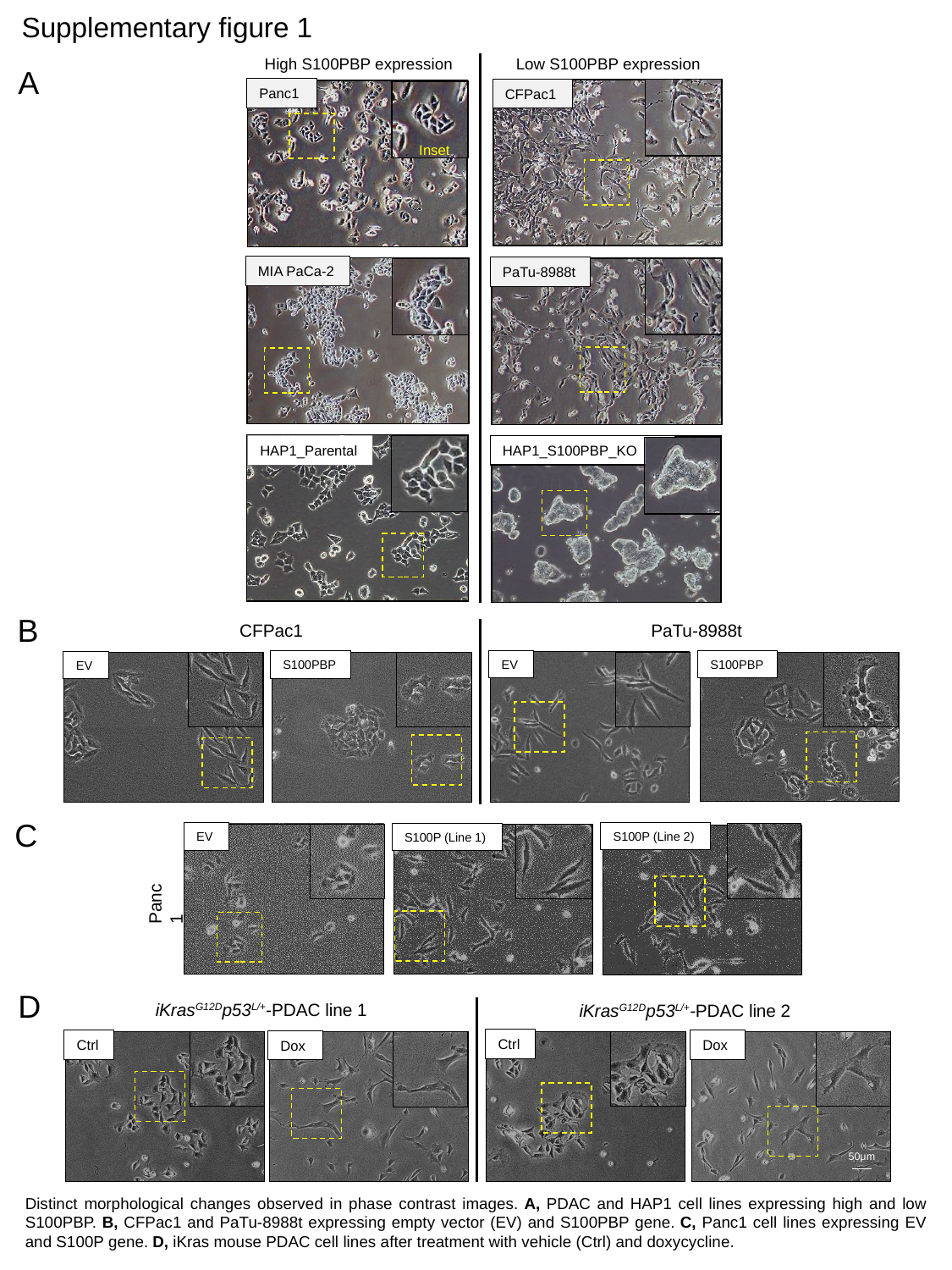

Supplementary figure 1
Low S100PBP expression
High S100PBP expression
Panc1
CFPac1
Inset
MIA PaCa-2
PaTu-8988t
HAP1_Parental
HAP1_S100PBP_KO
A
B
PaTu-8988t
CFPac1
EV
S100PBP
S100PBP
EV
C
S100P (Line 2)
EV
S100P (Line 1)
Panc1
D
iKrasG12Dp53L/+-PDAC line 1
iKrasG12Dp53L/+-PDAC line 2
Ctrl
Dox
Ctrl
Dox
50μm
Distinct morphological changes observed in phase contrast images. A, PDAC and HAP1 cell lines expressing high and low S100PBP. B, CFPac1 and PaTu-8988t expressing empty vector (EV) and S100PBP gene. C, Panc1 cell lines expressing EV and S100P gene. D, iKras mouse PDAC cell lines after treatment with vehicle (Ctrl) and doxycycline.

## Slide 2
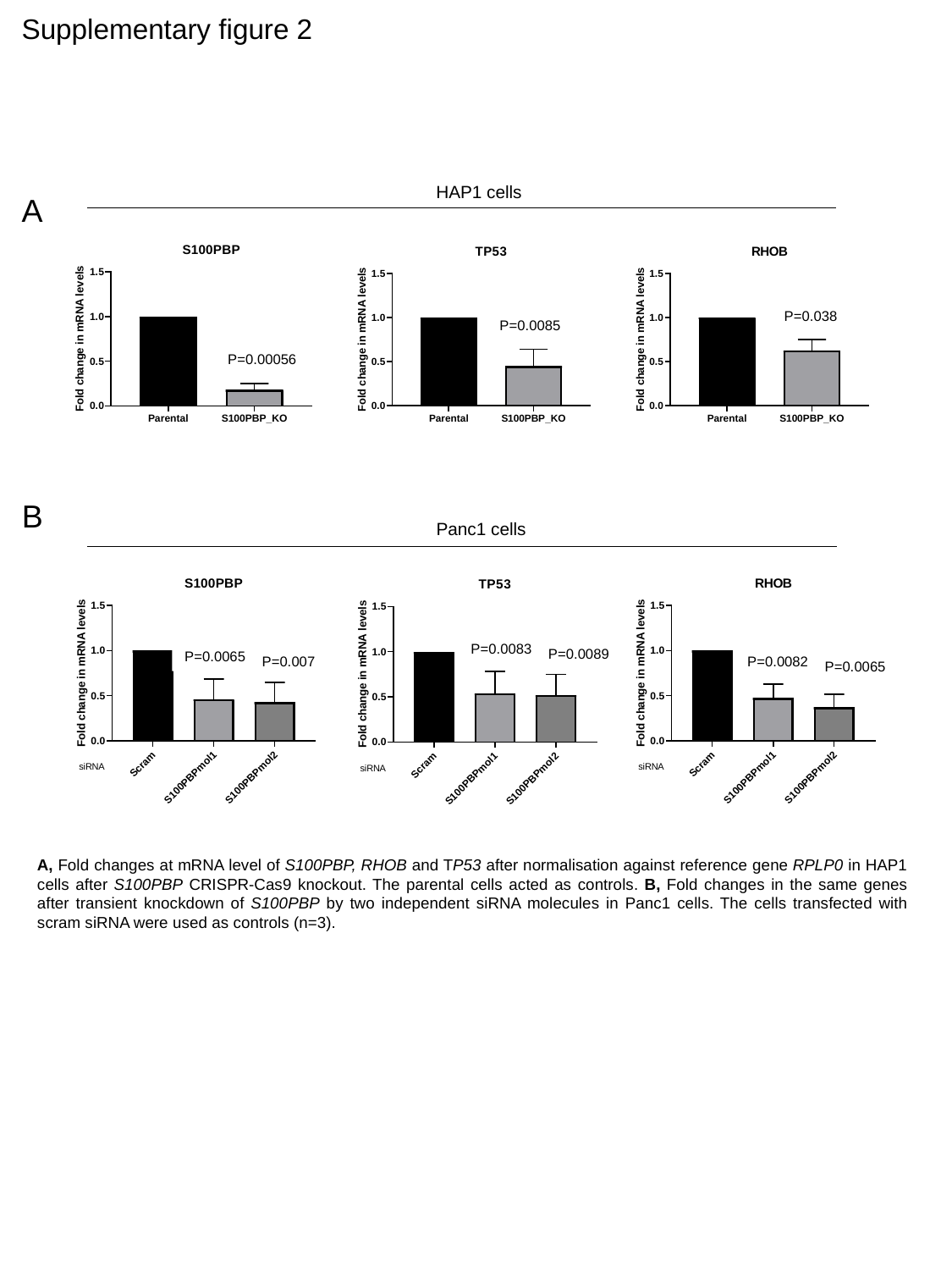

Supplementary figure 2
HAP1 cells
A
P=0.038
P=0.0085
P=0.00056
B
Panc1 cells
P=0.0083
P=0.0089
P=0.0065
P=0.0082
P=0.007
P=0.0065
A, Fold changes at mRNA level of S100PBP, RHOB and TP53 after normalisation against reference gene RPLP0 in HAP1 cells after S100PBP CRISPR-Cas9 knockout. The parental cells acted as controls. B, Fold changes in the same genes after transient knockdown of S100PBP by two independent siRNA molecules in Panc1 cells. The cells transfected with scram siRNA were used as controls (n=3).

## Slide 3
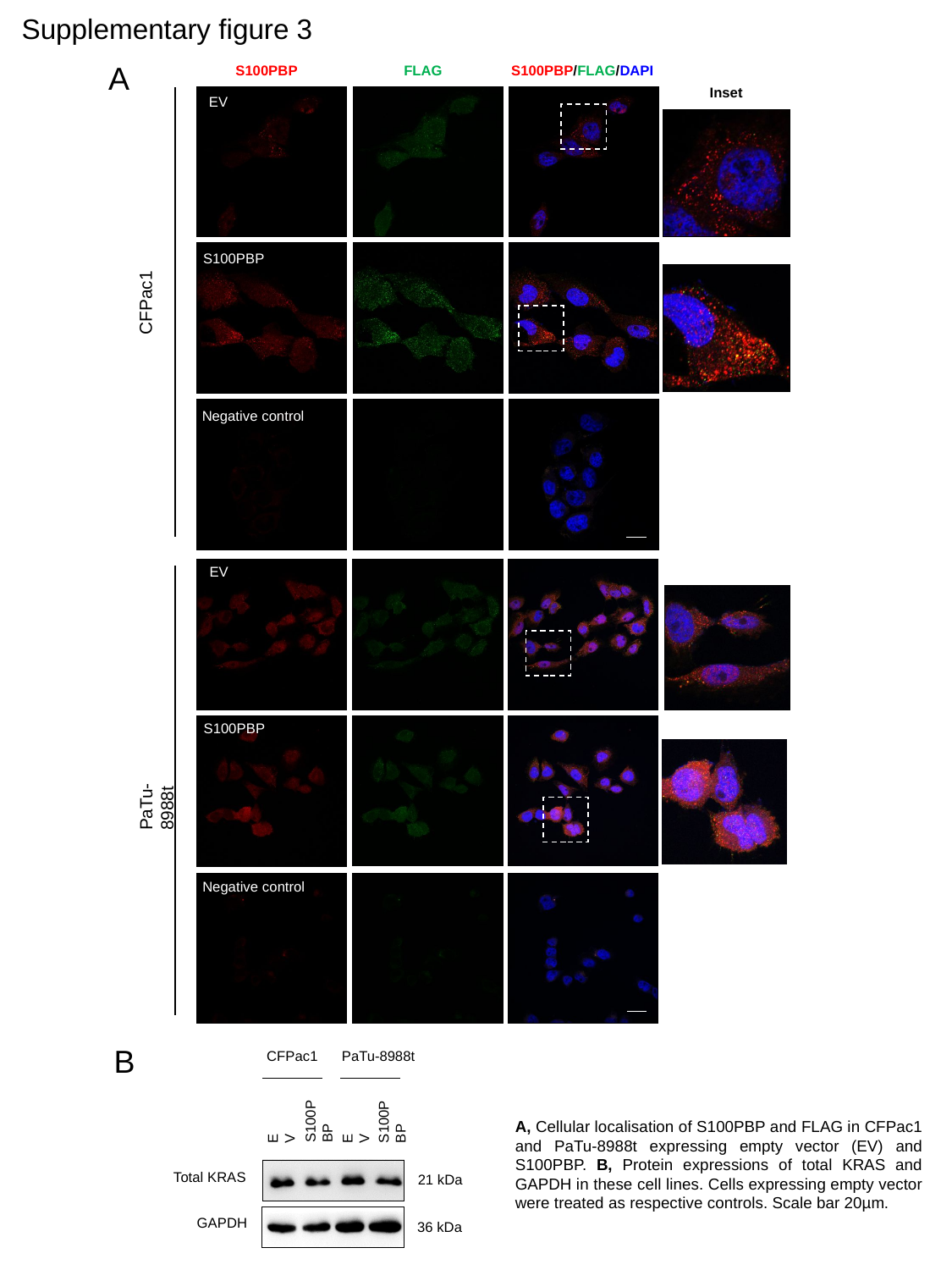

Supplementary figure 3
A
FLAG
S100PBP/FLAG/DAPI
S100PBP
Inset
EV
S100PBP
CFPac1
Negative control
EV
S100PBP
PaTu-8988t
Negative control
B
PaTu-8988t
CFPac1
S100PBP
S100PBP
EV
EV
Total KRAS
21 kDa
GAPDH
36 kDa
A, Cellular localisation of S100PBP and FLAG in CFPac1 and PaTu-8988t expressing empty vector (EV) and S100PBP. B, Protein expressions of total KRAS and GAPDH in these cell lines. Cells expressing empty vector were treated as respective controls. Scale bar 20µm.

## Slide 4
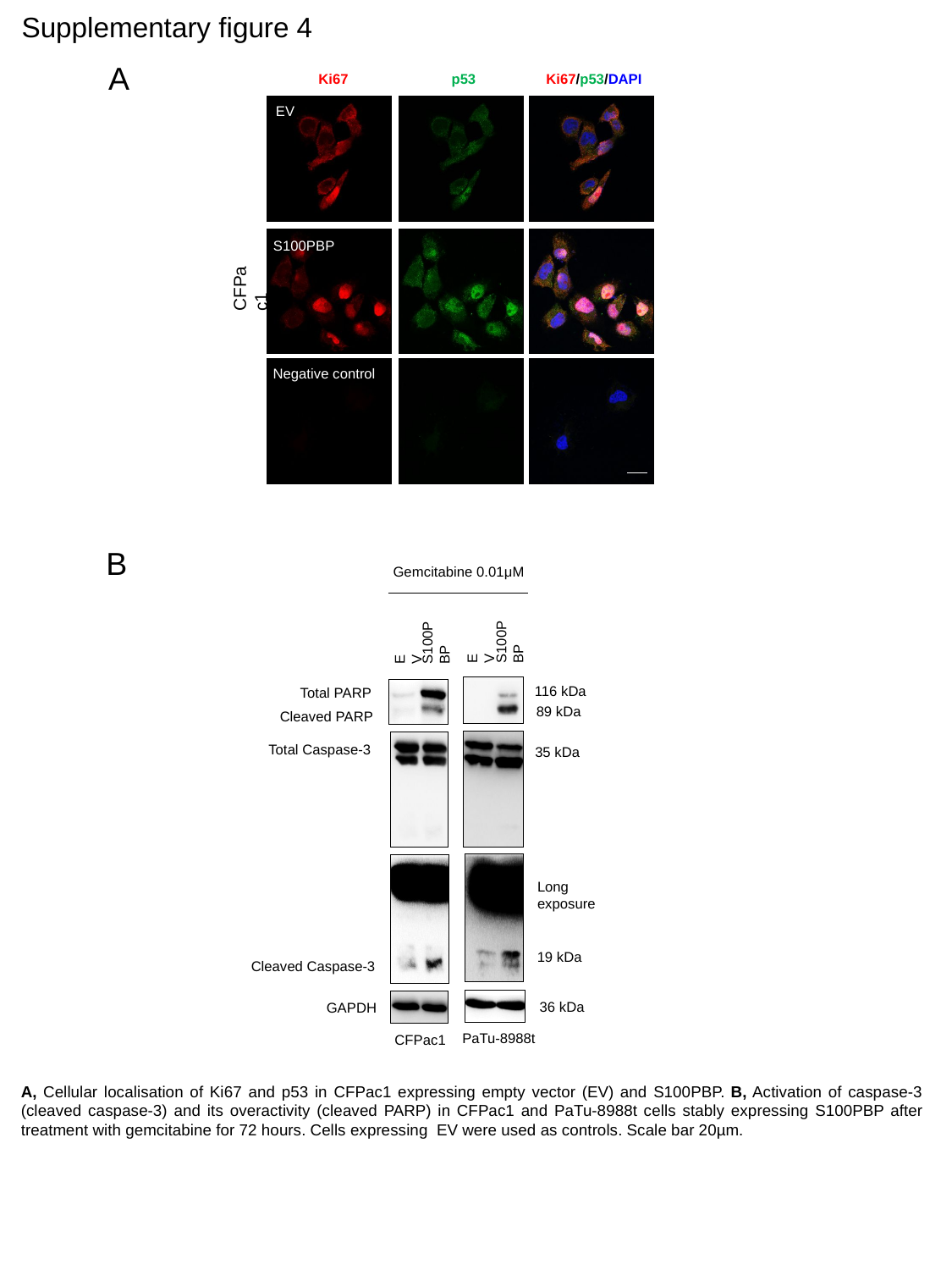

Supplementary figure 4
A
p53
Ki67/p53/DAPI
Ki67
EV
S100PBP
CFPac1
Negative control
B
Gemcitabine 0.01μM
S100PBP
S100PBP
EV
EV
116 kDa
Total PARP
89 kDa
Cleaved PARP
Total Caspase-3
35 kDa
Long exposure
19 kDa
Cleaved Caspase-3
36 kDa
GAPDH
PaTu-8988t
CFPac1
A, Cellular localisation of Ki67 and p53 in CFPac1 expressing empty vector (EV) and S100PBP. B, Activation of caspase-3 (cleaved caspase-3) and its overactivity (cleaved PARP) in CFPac1 and PaTu-8988t cells stably expressing S100PBP after treatment with gemcitabine for 72 hours. Cells expressing EV were used as controls. Scale bar 20µm.

## Slide 5
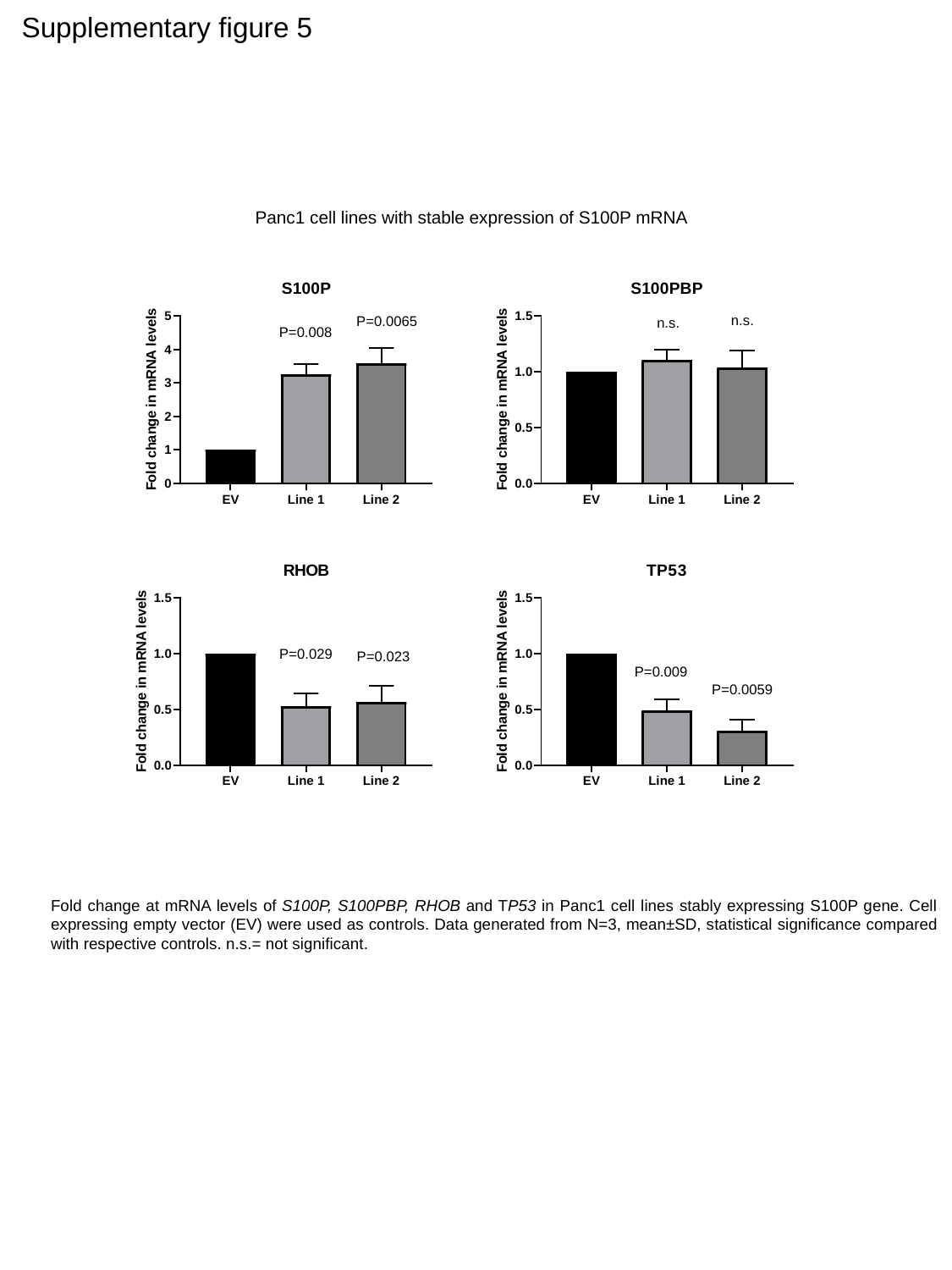

Supplementary figure 5
Panc1 cell lines with stable expression of S100P mRNA
n.s.
P=0.0065
n.s.
P=0.008
P=0.029
P=0.023
P=0.009
P=0.0059
Fold change at mRNA levels of S100P, S100PBP, RHOB and TP53 in Panc1 cell lines stably expressing S100P gene. Cell expressing empty vector (EV) were used as controls. Data generated from N=3, mean±SD, statistical significance compared with respective controls. n.s.= not significant.

## Slide 6
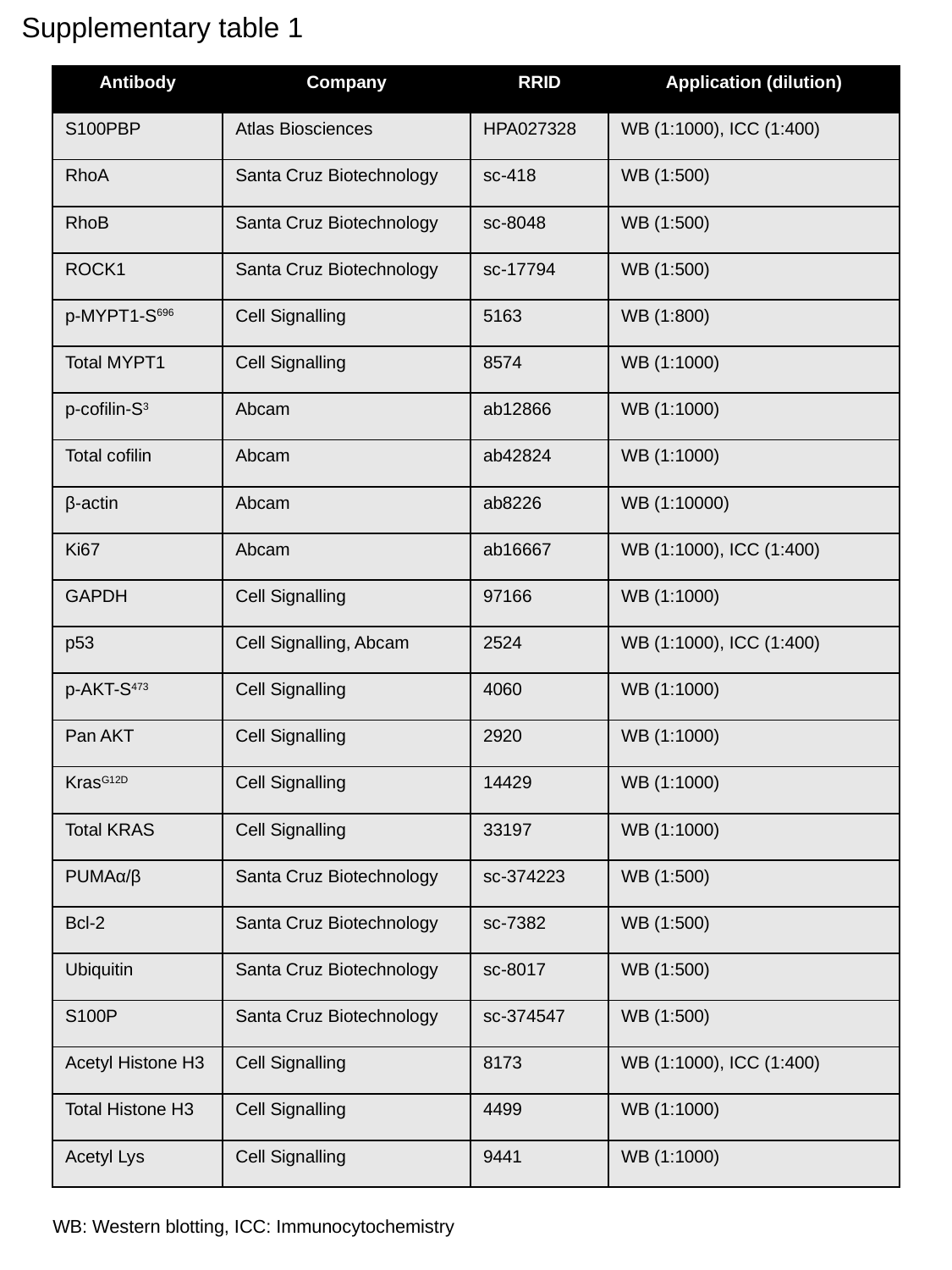

Supplementary table 1
| Antibody | Company | RRID | Application (dilution) |
| --- | --- | --- | --- |
| S100PBP | Atlas Biosciences | HPA027328 | WB (1:1000), ICC (1:400) |
| RhoA | Santa Cruz Biotechnology | sc-418 | WB (1:500) |
| RhoB | Santa Cruz Biotechnology | sc-8048 | WB (1:500) |
| ROCK1 | Santa Cruz Biotechnology | sc-17794 | WB (1:500) |
| p-MYPT1-S696 | Cell Signalling | 5163 | WB (1:800) |
| Total MYPT1 | Cell Signalling | 8574 | WB (1:1000) |
| p-cofilin-S3 | Abcam | ab12866 | WB (1:1000) |
| Total cofilin | Abcam | ab42824 | WB (1:1000) |
| β-actin | Abcam | ab8226 | WB (1:10000) |
| Ki67 | Abcam | ab16667 | WB (1:1000), ICC (1:400) |
| GAPDH | Cell Signalling | 97166 | WB (1:1000) |
| p53 | Cell Signalling, Abcam | 2524 | WB (1:1000), ICC (1:400) |
| p-AKT-S473 | Cell Signalling | 4060 | WB (1:1000) |
| Pan AKT | Cell Signalling | 2920 | WB (1:1000) |
| KrasG12D | Cell Signalling | 14429 | WB (1:1000) |
| Total KRAS | Cell Signalling | 33197 | WB (1:1000) |
| PUMAα/β | Santa Cruz Biotechnology | sc-374223 | WB (1:500) |
| Bcl-2 | Santa Cruz Biotechnology | sc-7382 | WB (1:500) |
| Ubiquitin | Santa Cruz Biotechnology | sc-8017 | WB (1:500) |
| S100P | Santa Cruz Biotechnology | sc-374547 | WB (1:500) |
| Acetyl Histone H3 | Cell Signalling | 8173 | WB (1:1000), ICC (1:400) |
| Total Histone H3 | Cell Signalling | 4499 | WB (1:1000) |
| Acetyl Lys | Cell Signalling | 9441 | WB (1:1000) |
WB: Western blotting, ICC: Immunocytochemistry
