# Supplementary material for: S100PBP is regulated by mutated KRAS and plays a tumour suppressor role in pancreatic cancer
Source: Oncogene. 2023 Oct 4;42(46):3422–34. doi: 10.1038/s41388-023-02851-y (PMC10638088; doi:10.1038/s41388-023-02851-y)
Supplement: Supplementary file 2 — Supplementary Table 2 [file 41388_2023_2851_MOESM2_ESM.pptx]

## Slide 1
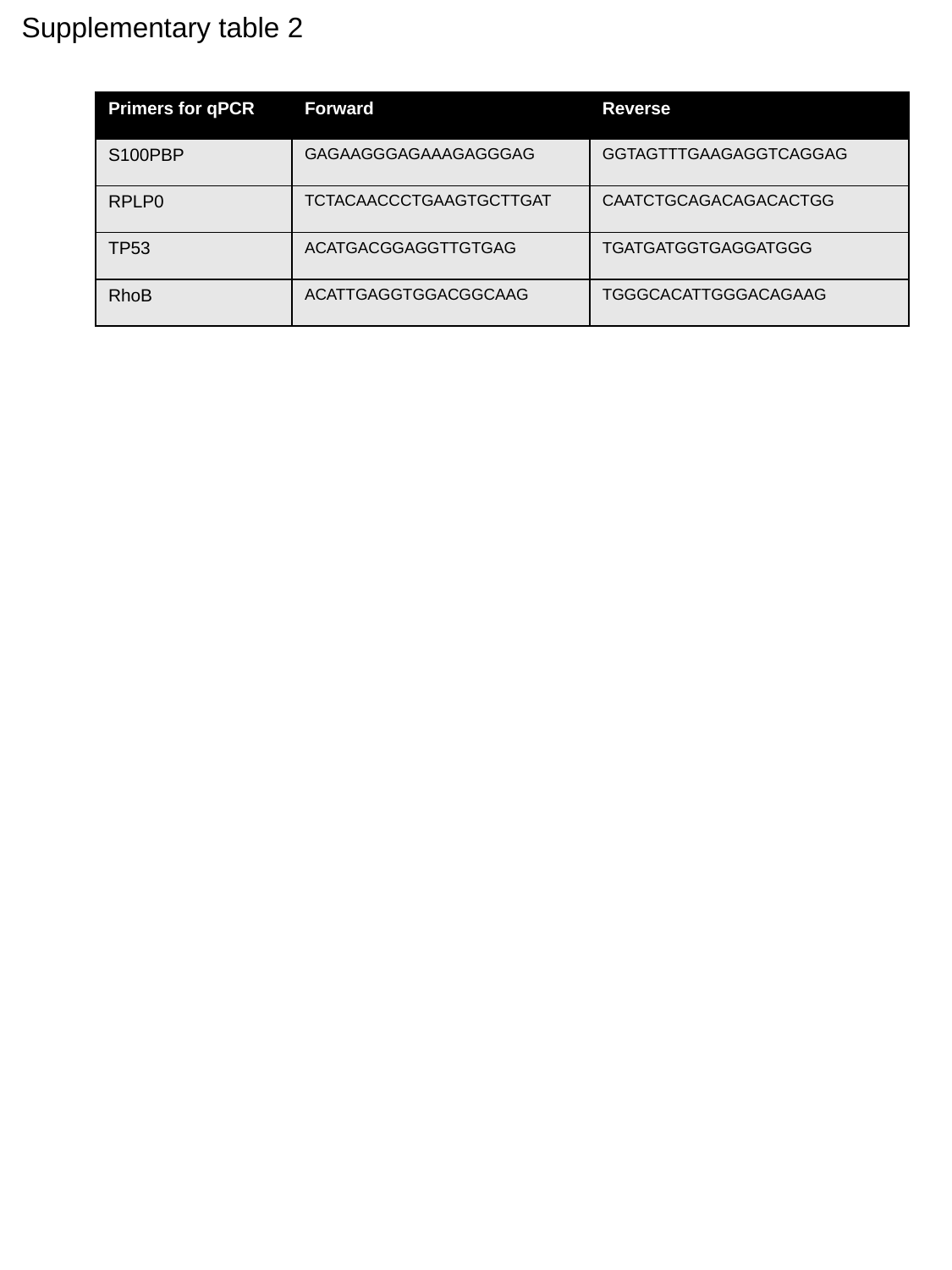

Supplementary table 2
| Primers for qPCR | Forward | Reverse |
| --- | --- | --- |
| S100PBP | GAGAAGGGAGAAAGAGGGAG | GGTAGTTTGAAGAGGTCAGGAG |
| RPLP0 | tctacaaccctgaagtgcttgat | Caatctgcagacagacactgg |
| TP53 | ACATGACGGAGGTTGTGAG | TGATGATGGTGAGGATGGG |
| RhoB | ACATTGAGGTGGACGGCAAG | TGGGCACATTGGGACAGAAG |
